# Factors associated with severe deep neck space infections: targeting multiple fronts

**DOI:** 10.1186/s40463-014-0035-5

**Published:** 2014-08-16

**Authors:** Brittany R Barber, Peter T Dziegielewski, Vincent L Biron, Andrew Ma, Hadi Seikaly

**Affiliations:** 1 Division of Otolaryngology-Head & Neck Surgery, University of Alberta, 1E4.29 Walter C Mackenzie Centre, 8440-112 Street NW, Edmonton, T6G 2B7 Alberta Canada; 2 Department of Otolaryngology, University of Florida, Gainesville, Florida USA; 3 Faculty of Medicine & Dentistry, University of Alberta, Edmonton, Canada

**Keywords:** Deep neck space infection, Odontogenic, Abscess, Airway obstruction, Socioeconomic status

## Abstract

**Objectives:**

To determine factors predictive of a severe deep neck space infection (DNSI), defined as those requiring surgery and/or postoperative intensive care unit (ICU) admission. To specifically examine dental practices and socioeconomic factors that may contribute to the development of a DNSI.

**Study design:**

Retrospective review.

**Methods:**

This study was conducted at 2 tertiary care academic referral centers from January 2007 to September 2011. The study was composed of 2 arms: a prospective questionnaire and data collection to identify modifiable risk factors such as dental practices and socioeconomic considerations for a DNSI, and a retrospective review of deep neck space infections to identify commonly associated risk factors predictive of a severe DNSI, requiring surgery and/or postoperative ICU admission.

**Results:**

233 patients were reviewed retrospectively and 25 patients prospectively. Patients with a low level of education (p = 0.03), those living greater than 1 hour from a tertiary care center (p = 0.002), those that have tonsils (p = 0.03), and those with *Streptococcus* infections (p = 0.03) have an increase risk of developing a severe DNSI. Patients that were smokers (p = 0.02) or had diabetes (p = 0.02), and those that presented with airway compromise (p = 0.03) were more likely to have a prolonged hospital stay.

**Conclusions:**

Factors predictive of severe DNSIs are *Streptococcus* infections, the presence of tonsils, education level, and geographic location.

## Introduction

Deep neck space infections (DNSIs) occur in potential spaces of the neck bound by cervical fascia. They can spread along fascial planes, and thus, may escalate quickly. During the pre-antibiotic era, most DNSIs were commonly precipitated by peritonsillar infections. In recent decades the most common infection source is thought to be odontogenic. Other cited risk factors include recent dental surgery, neck trauma, intravenous drug use (IVDU), and tobacco use [1].

Complications resulting from DNSIs are usually due to a delay in treatment, and often mandate surgery and/or a postoperative intensive care unit (ICU) admission. These may include upper airway obstruction, jugular venous thrombosis, descending mediastinitis, septic shock, and death [2],[3].

Although it has been reported that the overall incidence of DNSIs has been abated by the widespread use of antibiotics, severe DNSIs requiring urgent surgery, postoperative ICU treatment or prolonged hospital stays continue to be a common otolaryngologic emergency in many centers. By determining factors associated with severe DNSIs, potential morbidity may be decreased.

The aim of this study was to identify risk factors associated with severe DNSI, requiring operative intervention, ICU admission, or prolonged hospital stay. The first part of the study consisted of a prospective single cohort with a goal of identifying and quantifying modifiable risk factors, including dental practices and socioeconomic considerations, for a severe DNSI. The second part consisted of a retrospective review to re-examine the known risk factors for DNSI and determine if they have changed in the past 5 years.

## Materials and methods

Institutional review board ethics approval was granted by the University of Alberta *Health Research Ethics Board* (HREB) committee (Pro00009165). All patients involved in the prospective arm of the study provided informed, signed consent as per HREB guidelines.

### Setting and study design

This study was conducted at the University of Alberta’s 2 tertiary care academic referral centers (University of Alberta Hospital and Royal Alexandra Hospital) from January 2007 to September 2011.

This study included 2 parts: (I) a prospective cohort including all new DNSI referrals to the Otolaryngology-Head and Neck Surgery (Oto-HNS) service at the University of Alberta from 2011–2012 as well as (II) a retrospective group of all DNSIs treated by the Oto-HNS service at the University of Alberta from 2007–2011.

### Research questions

1. What factors are associated with severe DNSIs, defined by the following:
  - What factors are associated with DNSIs needing an open incision and drainage?
  - What factors are associated with DNSIs needing an ICU stay?
  - What factors are associated with DNSIs needing prolonged hospital stay?
2. What are the common bacteria cultured in severe DNSIs?
3. Have the risk factors for severe DNSI in Northern Alberta changed over the last 5 years?

### Patient selection and data collection

#### (I) Prospective cohort

A prospective cohort was utilized to identify risk factors associated with severe DNSI, which could not otherwise be gleaned from a retrospective review. This included standard demographic information as well as dental practices and socioeconomic data. All patients with a DNSI referred from January 1, 2011 – January 1, 2012 to the Oto-HNS service at the University of Alberta were considered for study enrollment.

Inclusion criteria consisted of:

1. Age ≥ 18 years
2. Diagnosis of DNSI by computed tomography (CT). This included a phlegmon, ring enhancing abscess or cervical necrotizing fasciitis as reported by the attending radiologist on call.

Exclusion criteria consisted of:

1. DNSI confined to the peri-tonsillar space
2. DNSI in the investing fascia (i.e. a superficial neck abscess)
3. DNSI confined to a dentoalveolar space

Patients meeting enrolment criteria had the study explained to them in detail by a designated Oto-HNS resident on call. They were also required to read over a study information sheet prior to signing informed consent. Patients were either enrolled in the emergency department prior to hospital admission, or if requiring urgent surgery, were enrolled during their hospital stay. Once enrolled, patients were asked to complete a questionnaire. Questionnaires were then collected by the Oto-HNS trial coordinator and the data was compiled into a prospective database.

Additional data collected included: age, gender, airway status at presentation, season of presentation, space involvement, treatment, ICU stay, hospital stay, as well as culture results. The treatment strategy for all patients was standardized as per previously described departmental guidelines [4]. Patients with an unstable airway were taken to the operating room to have their airway secured. Those with an identifiable abscess on CT scanning underwent an incision and drainage. Patients with a deep neck phlegmon were started on intravenous (IV) broad-spectrum antibiotics and re-assessed in 24-48 hrs. If there was no clinical improvement in signs and symptoms, those patients were taken to the OR for an incision and drainage.

The primary outcome was the identification of modifiable risk factors for the development of severe DNSI. Markers of a severe DNSI included: an operative incision and drainage, ICU stay and length of hospital stay (LOS) > 3 days.

#### (II) Retrospective review

A retrospective review was performed to provide robust power for analyzing standard demographic factors potentially associated with severe DNSI. An electronic medical records search was conducted of all patients with a possible DNSI from 2007–2011. The search strategy included: “deep neck space infection”, “neck space infection”, “neck infection”, “neck abscess”, “cervical necrotizing fasciitis”, “deep neck” and “neck swelling”. Other terms were used to ensure inclusion of all appropriate diagnoses (“dental infection”, “odontogenic infection”, “cervical infection”), but discarded if the described lesion was a dentoalveolar abscess, peritonsillar abscess, or did not include phlegmon or abscess of the tissues deep to the investing fascia. Data points collected included age at diagnosis, gender, presenting complaint, site of infections as per CT, assumed origin, intervention, length of hospital stay, whether the patient required an admission to the (ICU), and whether the patient suffered any additional severe complications such as an emergent tracheostomy, jugular venous thrombosis, descending mediastinitis, or death. Presence of risk factors, including recent dental surgery, tobacco use, marijuana use, alcohol use, intravenous drug use, diabetes, Hepatitis B/C, HIV, and other immunocompromised states was also recorded.

### Statistical analysis

All potential risk factors for DNSIs were analyzed using correlation analysis. Chi-squared/Fisher exact test analysis was used for all potential risks factors to determine factors associated with the need for operative intervention, ICU admission, and prolonged LOS. Statistical analysis was conducted using SPSS 17.0 (SPSS Inc., Chicago, IL).

### Ethics approval

Ethics approval was granted by the University of Alberta’s *Health Research Ethics Board*. Informed consent was obtained from each patient for data usage.

## Results

### (I) Prospective analysis

Over 12 months, 25 questionnaires were completed in consecutive patients with DNSIs. The median age of participants was 43.5 years (range: 18–82 years) with most presenting during the winter months (64%). The most common microbe isolated from surgical specimens or aspirates was *Streptococcus anginosus* (40%), followed by *Streptococcus pyogenes* (12%) and *Staphylococcus aureus* (12%). Table 1 demonstrates clinical, dental and socioeconomic characteristics of the cohort. Most patients did not have significant comorbidities. Two patients had type 2 diabetes mellitus, 1 had Hepatitis C, 1 had HIV, and 4 were hypothyroid. There was only 1 intravenous drug user, who did not inject into neck vessels.

**Table 1:** **Patient characteristics and their associations with indicators of severe DNSI**

Clinical characteristic	N (%)	OR I&D	Post-op ICU	LOS > 3d
Age > 55 years		*0.50*	*0.45*	*0.12*
< 55 years	18 (72)	12 (67)	8 (44)	14 (78)
> 55 years	7 (28)	4 (57)	4 (57)	3 (43)
Gender		*0.41*	*0.16*	*0.08*
Males	16 (64)	11 (69)	6 (67)	13 (82)
Females	9 (36)	5 (56)	6 (38)	4 (44)
Race		*0.69*	*0.62*	*0.68*
Caucasian	22 (88)	14 (64)	11 (50)	15 (68)
First Nations	2 (8)	1 (50)	1 (50)	1 (50)
East Indian	1 (4)	1 (100)	0 (0)	1 (100)
Smoking status		*0.79*	*0.43*	***0.04***
Non-smoker	13 (52)	9 (69)	6 (46)	7 (54)
Smoker	12 (48)	7 (58)	6 (50)	10 (83)
Alcohol consumption		*0.39*	*0.46*	*0.53*
Non-daily	20 (80)	12 (60)	9 (45)	14 (70)
Daily	5 (20)	4 (80)	3 (60)	3 (60)
Season of presentation		*0.26*	*0.44*	*0.37*
Non-winter	9 (36)	7 (78)	5 (56)	7 (78)
Winter	16 (64)	9 (56)	7 (44)	10 (63)
Airway status on presentation		***<0.001***	***0.03***	*0.13*
No distress	10 (40)	2 (20)	2 (20)	5 (50)
In distress	15 (60)	14 (93)	10 (67)	12 (80)
Deep neck spaces involved		*0.64*	*0.64*	*0.27*
Single	19 (76)	12 (63)	9 (47)	14 (74)
Multiple	6 (24)	4 (67)	3 (50)	3 (50)
Microbiology		***0.03***	*0.42*	*0.08*
*Strep*. species	13 (52)	11 (85)	7 (54)	6 (50)
Other	12 (48)	5 (42)	5 (42)	11 (85)

*Abbreviations:* N, number; OR, operating room; I&D, incision and drainage; Post-op, post-operative; ICU, intensive care unit; LOS, length of hospital stay; d, days.

Legend: brackets, percentage; bold, statistical significance.

Data given as: *p-value.*

number (percent).

Table 2 demonstrates the oral health characteristics and habits of the prospective cohort. The questionnaires elucidated that most patients consistently brushed their teeth; however, flossing and yearly dental visits were not common habits.

**Table 2:** **Oral health characteristics and their associations with indicators of severe DNSI**

Oral health characteristic	N (%)	OR I&D	Post-op ICU	LOS > 3d
Tooth status		0.71	0.47	0.70
Teeth present	22 (88)	14 (64)	10 (46)	15 (68)
Edentulous	3 (12)	2 (67)	2 (67)	2 (68)
Tonsil status		**0.03**	0.55	0.12
Absent	7 (28)	2 (29)	3 (43)	3 (43)
Present	18 (72)	12 (78)	9 (50)	14 (78)
Tooth brushing		0.29	0.12	0.70
Non-daily	3 (12)	1 (33)	0 (0)	2 (67)
Daily	22 (88)	15 (68)	12 (55)	15 (68)
Dental flossing		0.39	0.54	0.48
Non-daily	20 (80)	12 (60)	10 (50)	13 (65)
Daily	5 (20)	4 (80)	2 (40)	4 (80)
Dental visits		0.33	0.82	0.50
Less than yearly	14 (56)	10 (71)	7 (50)	10 (72)
Yearly or more	11 (44)	6 (55)	5 (46)	7 (64)
Recent dental procedure		0.06	0.44	0.37
None	16 (64)	8 (50)	7 (44)	10 (63)
Within 2 weeks of DNSI	9 (36)	8 (89)	5 (56)	7 (78)

*Abbreviations:* N, number; OR, operating room; I&D, incision and drainage; Post-op, post-operative; ICU, intensive care unit; LOS, length of hospital stay; d, days; DNSI, deep neck space infection.

Legend: brackets, percentage; bold, statistical significance.

Data given as: *p-value.*

number (percent).

Table 3 shows the socioeconomic characteristics of the prospective cohort. The median income was $30000 (range: $0 - $170000). 52% of the patients were subsisting on an annual income below the established poverty line for a four-person family living in Canada [5]. Most patients completed high school and 20% even had post-secondary degrees.

**Table 3:** **Socioeconomic characteristics and their associations with indicators of severe DNSI**

Socioeconomic characteristic	N (%)	OR I&D	Post-op ICU	LOS > 3d
Yearly income (CDN)		0.56	0.32	0.29
< $20 000	12 (48)	8 (67)	7 (58)	7 (58)
> $20 000	13 (52)	8 (62)	5 (39)	10 (77)
Education level		0.26	**0.03**	0.37
Completed < high school	9 (36)	7 (78)	7 (78)	7 (78)
Completed high school	16 (64)	9 (56)	5 (31)	10 (63)
Home location		0.41	0.25	0.29
Rural	9 (36)	5 (56)	3 (33)	5 (56)
Urban	16 (64)	11 (69)	9 (56)	12 (75)

*Abbreviations:* N, number; OR, operating room; I&D, incision and drainage; Post-op, post-operative; ICU, intensive care unit; LOS, length of hospital stay; d, days; CDN, Canadian.

Legend: brackets, percentage; bold, statistical significance.

Data given as: *p-value.*

number (percent).

### (II) Retrospective analysis

The electronic search yielded 432 potential cases. After the exclusion criteria were applied, there were 233 DNSIs that were included in the study. The mean age of the patients involved was 42.6 years (range: 18–104) with most (54%) presenting in the non-winter months. The average number of DNSIs per year at the University of Alberta was 49 for a catchment area of 1.8 million.

In terms of management strategies, 53% of patients required an incision and drainage for definitive treatment of their DNSI, whereas 8.5% were treated with an ultrasound-guided needle aspiration. 21.4% required incision and drainage as well as postoperative ICU admission, and 2.9% required an incision and drainage and tracheostomy. 11.4% of patients were treated with intravenous antibiotics only. Therefore, 77.3% of patients required an operation for resolution of their DNSI.

Table 4 demonstrates patient characteristic details and their associations with markers of severe DNSIs.

**Table 4:** **Patient characteristics and their associations with indicators of severe DNSI (retrospective review)**

Characteristic	N (%)	OR I&D (p-value)	Post-op ICU (p-value)	LOS (p-value)
Age > 55 years		0.94	0.35	0.89
< 55 years	120 (79)	87 (73)	32 (27)	87 (73)
> 55 years	32 (21)	23 (72)	11 (36)	23 (72)
Gender		0.29	0.15	0.12
Males	104 (68)	78 (75)	33 (32)	79 (77)
Females	48 (32)	32 (67)	10 (21)	31 (65)
Smoking status		0.11	0.53	**0.02**
Non-smoker	40 (36)	27 (61)	9 (21)	24 (60)
Smoker	71 (64)	63 (77)	21 (26)	62 (75)
Alcohol consumption		0.57	0.51	0.59
Non-daily	74 (75)	55 (75)	16 (22)	48 (65)
Daily	25 (25)	20 (80)	7 (28)	17 (71)
Intravenous drug use (IVDU)		0.19	0.84	0.53
None	86 (78)	61 (71)	19 (22)	55 (64)
Current	24 (22)	21 (84)	6 (24)	17 (71)
DM2		0.13	0.40	**0.02**
No	133 (88)	99 (75)	36 (28)	92 (70)
Yes	19 (12)	11 (58)	7 (37)	18 (95)
Season of presentation		0.33	0.41	0.81
Non-Winter	82 (54)	82 (75)	33 (31)	80 (73)
Winter	70 (46)	28 (67)	10 (24)	30 (71)
Airway status at presentation		**0.03**	**<0.001**	**0.03**
Non-Compromised Airway	19 (12)	18 (95)	27 (21)	18 (95)
Compromised Airway	133 (88)	92 (69)	16 (84)	92 (70)
Suspected source of Infection		0.37	0.28	0.36
Non-odontogenic	81 (53)	56 (69)	26 (61)	60 (76)
Odontogenic	71 (47)	54 (76)	17 (24)	49 (69)
Geographic location		0.19	**0.002**	0.19
Urban	145 (95)	103 (71)	37 (26)	102 (71)
Rural	7 (5)	7 (100)	6 (86)	7 (100)
Homeless		0.67	0.67	0.61
Not Homeless	143 (95)	104 (72)	42 (30)	104 (73)
Homeless	7 (5)	6 (86)	1 (14)	5 (72)

*Abbreviations:* N, number; OR, operating room; I&D, incision and drainage; Post-op, post-operative; ICU, intensive care unit; LOS, length of hospital stay; d, days; IV, intravenous; DM2, type 2 diabetes mellitus.

Legend: brackets, percentage; bold, statistical significance.

Data given as: *p-value.*

number (percent).

## Discussion

Deep neck space infections (DNSIs) continue to challenge Otolaryngologists from a medical, surgical, and socioeconomic perspective. Although the advent of modern antibiotics has allowed for uncomplicated treatment of milder DNSIs, severe infections often manifest with airway compromise necessitating urgent surgical intervention and postoperative ICU admission. Our results reflect this point appropriately. Our study also demonstrated that geographic location, education level, tonsil status, and microbiological characteristics are associated with severity of DNSIs.

The microbiological characteristics of DNSIs have been studied extensively in the past 15 years as resistance rates to conventional antibiotics have become a concern. Common culprits for DNSIs include the *Streptococcus anginosus* group (SAG), (including *Streptococccus anginosus*, *Streptococcus intermedius*, and *Streptococcus constellatus*), *Streptococcus pyogenes*, *Streptococcus viridans*, *Staphylococcus aureus,* and multiple species of anaerobes. The *SAG* microbes have a propensity for forming abscesses and causing invasive pyogenic infection in the head and neck, brain, intrathoracic, and intra-abdominal region due to the production of extracellular enzymes capable of degrading connective tissue. This microbial assembly is also known to release extracellular products, which have an immunosuppressive effect, allowing survival within the walls of an abscess. The SAG microbes are the culprit in a large percentage of dental abscesses, and thus, are the responsible microbe in a large percentage of DNSIs, as is consistent with our findings of 35.9% in the prospective group and 21.1% in the retrospective group. It follows that *Streptococcus* species were a significant contributor in predicting those patients requiring surgical intervention in our cohort. This finding is supported by previous studies, which demonstrate a prevalence of *Streptococcus* species in DNSIs requiring surgical intervention for resolution [6]-[9].

Previously established risk factors for DNSIs have included peritonsillar infections, upper respiratory tract infections, poor oral health, odontogenic infection, intravenous drug use (IVDU) or other substance abuse, immunocompromised states, and diabetes [10]. More recent trends have demonstrated an overall increase in the prevalence of cases related to odontogenic infection [11]. As such, this study was designed to examine oral health characteristics, in patients presenting with DNSIs, which may predispose to severe DNSI. Interestingly, it was found that teeth-brushing, flossing, regular dental visits, and recent dental procedures were not predictors of severe DNSIs. This could be a result of the infections being caused by more virulent bacteria, which may be more resistant to standard dental practices. Secondly, the patient population may have reflected a cohort less likely to present to a health practitioner at an earlier stage of infection.

Having tonsils was also found to be associated with severe DNSI, specifically the need for operative intervention. This could be a result of an increased bacterial load harbored within the tonsillar crypts. This study is the first, to the authors’ knowledge, to describe an association between the presence of tonsils and severe DNSI.

Interestingly, patients that had an annual income of less than $20000 per year were not found to have more severe DNSIs, inferring that affordability of dental care may not have been a contributing factor in presenting patients. However, affordability of dental care would only have been a factor in 47% of the cohort, which presented with likely odontogenically-sourced DNSIs. Furthermore, homeless patients, who had no access to dental care, were also not more likely to develop a severe DNSI. In contrast, education level (those patients achieving less than a high-school education) was shown largely to be of predictive value for postoperative ICU admission. This could have reflected a lack of health awareness leading to delayed presentation with a larger degree of airway compromise necessitating prolonged ICU care. Socioeconomic status (SES) and its association with DNSIs was examined in a recent study by Agarwal et al. [12], which demonstrated that, of 120 patients with DNSIs presenting to a tertiary care center, 90% were of low SES according to the revised Kuppuswamy scale [13], 70% were illiterate, and 0% were aware of any of the predisposing factors and potential complications of DNSIs. These findings further highlight that socioeconomic disparities lead to increasingly severe presentation of DNSI.

Patients that presented to a tertiary care center from a geographic location greater than 1 hour away were found to have more severe DNSIs, which necessitated postoperative ICU admission. Remote access to health care could have lead to delayed presentation, and thus, resulted in a greater degree of airway compromise requiring critical interventions. In addition, it is postulated that limited access to tertiary care prohibited appropriate abortive treatment at the primary care level.

In terms of length of hospital stay, this study demonstrated that presentation with airway compromise, tobacco use, and diabetes are associated with a prolonged course in hospital, as is consistent with the findings of previous studies [14]-[17].

Not only are the severity and potential for complications of DNSIs alarming, but the economic burden imposed on the healthcare system of surgical intervention and admission to intensive care settings are a concern. Biron et al. [4] evaluated cost-effectiveness in the management of DNSIs and found that hospital admission, surgical intervention, and a postoperative ICU admission for one night amount to $4629.57 in the Canadian healthcare system. This study also compared ultrasound-guided needle drainage (USD) and surgical incision and drainage in the operating room, and found a decrease in length of hospital stay for USD (3.1 vs 5.2 days), and an overall cost savings of $8809.68 per patient. Identifying patients that are medically stable and do not possess the risk factors for a severe DNSI may allow for the institution to pursue such cost-effective management strategies. Furthermore, given the potential for complications and economic ramifications of severe DNSIs, consideration should be given to identifying causality and instituting preventative measures. Identification and education regarding severity of DNSIs at the primary care level may allow for early prevention or intervention strategies to prevent the seemingly common presentation of airway compromise, necessitating surgical drainage and postoperative ICU admission. This is particularly important in rural areas where health care resources may be scarce.

## Conclusion

DNSIs, although common in Otolaryngology, can have severe complications. Identifying risk factors for a severe DNSIs can expedite necessary surgical management and appropriate postoperative care. Patients with low education, smokers, living > 1 hour from a tertiary care center, having tonsils or streptococcus infections, are at increased risk of severe DNSIs. Such patients with a DNSI should be targeted for early referral to an Otolaryngologist or expedited critical care management.

## Presentation

This study was presented at the Canadian Society of Otolaryngology-Head and Neck Surgery 65^th^ Annual Meeting in Victoria, BC on May 22–24, 2011.

Level of Evidence: 2c.
